# Membrane potential synchrony of neurons in anterior cingulate cortex plays a pivotal role in generation of neuropathic pain

**DOI:** 10.1038/s41598-018-20080-2

**Published:** 2018-01-26

**Authors:** Zhiyu Chen, Xiaolu Shen, La Huang, Hai Wu, Mazhong Zhang

**Affiliations:** 1 Department of Anesthesiology, Shanghai Children’s Medical Center, Shanghai Jiaotong University School of Medicine, Shanghai, China; 2 Department of Anesthesiology and Intensive Care Medicine, Xinhua Hospital, College of Medicine, Shanghai Jiaotong University, Shanghai, China; 3 Department of Surgery, Suining Central Hospital, Suining, China

## Abstract

The pathophysiology of neuropathic pain generation has not been fully investigated. Previous studies have primarily focused on changes in the properties of single neurons in the brain after nerve injury; however, little is known concerning the role of neuron-to-neuron connections in neuropathic pain pathogenesis. Synaptic transmission potentiation in anterior cingulate cortex (ACC) has been confirmed to be responsible for the formation of neuropathic pain. Thus, analysis of interneuronal connections in the ACC is an important approach for understanding the mechanism of neuropathic pain since it provides information on the potency of synaptic transmission. Here, we recorded membrane potentials from pairs of ACC neurons in anaesthetised rats and found that cross-correlations between pairs of ACC neurons significantly increased after surgery for chronic constriction injury (CCI). Moreover, CCI surgery could also enhance the power spectrum density of lower and higher-frequency membrane oscillations while having no effect on middle-frequency oscillations. The activation of membrane potential synchrony and power spectrum was reversed by the electrical synapse blocker mefloquine and pain behaviour was simultaneously alleviated. Our results may indicate that activation of membrane potential synchrony contributes to generation of neuropathic pain.

## Introduction

Neuropathic pain brings great suffering to patients and seriously affects the normal work and daily life of patients. Although management of neuropathic pain has evolved, the effectivenoess of treatment is often limited. After treatment with drugs that have specific indications for neuropathic pain, including pregabalin and duloxetine, only one-fourth of these patients find pain relief^1^. This is partially due to the fact that the underlying pathophysiology of neuropathic pain development has not been fully elucidated. Although many studies have confirmed that changes of neuronal properties at the single cell level in the brain after nerve injury are crucial for neuropathic pain formation^2,3^, little is known concerning the role that correlated activity between neurons plays in neuropathic pain pathogenesis. Indeed, a more complete understanding of the brain will come from recording activity from populations of neurons, rather than from one neuron at a time. Thus, to gain a better understanding of pathophysiology of neuropathic pain, we set out to study changes in the interneuronal correlations using dual patch clamp recording in a neuropathic pain model.

Interneuronal correlations have been found to play a key role in many cognitive functions, such as information transfer, perception, learning and attention^4–7^. The correlation of spontaneous membrane potential (V_m_) activities in neurons varies in different cognitive activities^5,8^. The V_m_ activity of neurons can be synchronised or desynchronised in neural information processing^6^. Interneuronal correlations of spontaneous membrane potential can give rise to oscillations at different frequencies^9^. Moreover, painful stimuli have been found to activate neuronal oscillations in the portion of the cortex associated with pain^10^. It has also been reported that oscillatory gamma activity is significantly enhanced under chronic pain conditions and that the power of oscillations is positively correlated with hyperalgesia of chronic pain^11^. Therefore, interneuronal correlations of V_m_ activity may participate in pain processing.

Our previous work found that electrical synapses have special roles in the pathophysiology of neuropathic pain and that the function of electrical synapses was enhanced an animal model of neuropathic pain^12^. Experiments have revealed that electrical synapses are involved in synchronising the activity of different neurons^13,14^. Changes in the function of electrical synapses in neuropathic pain may lead to the modification of the connections between different neurons. Thus, analysis of the membrane potential synchrony in neurons may also provide insight into the mechanisms by which electrical synapses generate neuropathic pain.

In the brain, the role of anterior cingulate cortex (ACC) in generation of neuropathic pain has received increasing attention. It has been demonstrated that ablation of ACC can largely alleviate pain without significantly affecting the sensory-discriminative perception of the stimuli^15^. Previous studies have also demonstrated that synaptic transmission enhancement in the ACC neurons contributes to the maintenance of neuropathic pain^16^. Thus, the ACC is a key cortical area involved in neuropathic pain. Furthermore, the high-frequency components of membrane potential synchrony have been proposed to control the timing of spikes and neural signal transduction^6,17^. Thus, analysis of the correlated of V_m_ activity in neurons of the cerebral cortex is an important method that provides information regarding the potency of synaptic transmission. The potency of synaptic transmission in the ACC plays a key role in pathogenesis of neuropathic pain^16^; therefore, interneuronal correlations in the ACC may play crucial role in neuropathic pain states. In the present study, using a method of *in vivo* dual patch clamp whole-cell recording from ACC neurons in anesthetized adult rats, we investigated the changes in membrane potential synchrony between pairs of neurons within 1 to 2 weeks after chronic constriction injury (CCI) surgery.

## Results

### Pain hypersensitivity induced by CCI surgery

**Figure 1:**
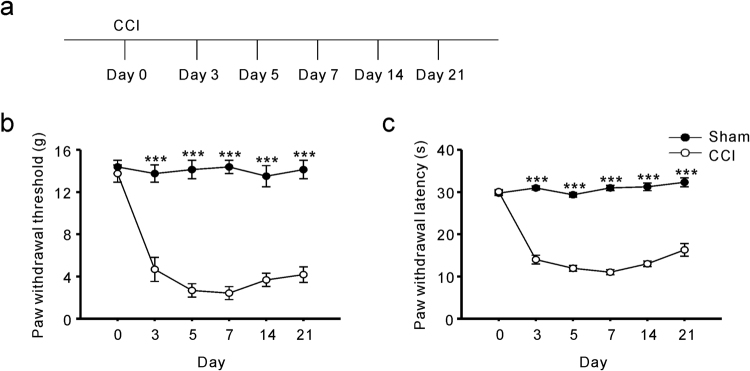
Pain hypersensitivity in chronic constriction injury (CCI) rats. (**a**) Schematic view of establishment of neuropathic pain animal model. (**b**) Thresholds for hindpaw withdrawal responses to von-Frey filament stimulation in sham and CCI rats (two-way ANOVA, ***P < 0.001, compared to sham group, n = 9). (**c**) Latencies of hindpaw withdrawal responses to a thermal stimulus in sham and CCI rats (two-way ANOVA, ***P < 0.001, compared to sham group, n = 9).

After CCI surgery was performed, we examined the development of neuropathic pain-like behaviours using mechanical allodynia tests and thermal hyperalgesia tests. Pain behaviors were investigated on days 0 (the day of surgery), 3, 5, 7, 14, and 21 after CCI (Fig. 1a). In our measurements from CCI rats, the paw withdrawal threshold to mechanical stimuli was significantly reduced to 4.7 ± 1.1 g (p < 0.001) on day 3 and further reduced to 2.4 ± 0.6 g (p < 0.001) on day 7, compared with the pain threshold measured in the sham group (14.4 ± 0.6 g) (Fig. 1b). In addition, all the CCI rats exhibited thermal hyperalgesia, which was indicated by a significantly shorter paw withdrawal latency to heat stimuli than that measured in the sham group (Fig. 1c). Pain hypersensitivity to both mechanical and thermal stimuli could lasted for at least 3 weeks after CCI (Fig. 1b and c).

### Increase in the cross-correlation of spontaneous membrane potential after CCI surgery

**Figure 2:**
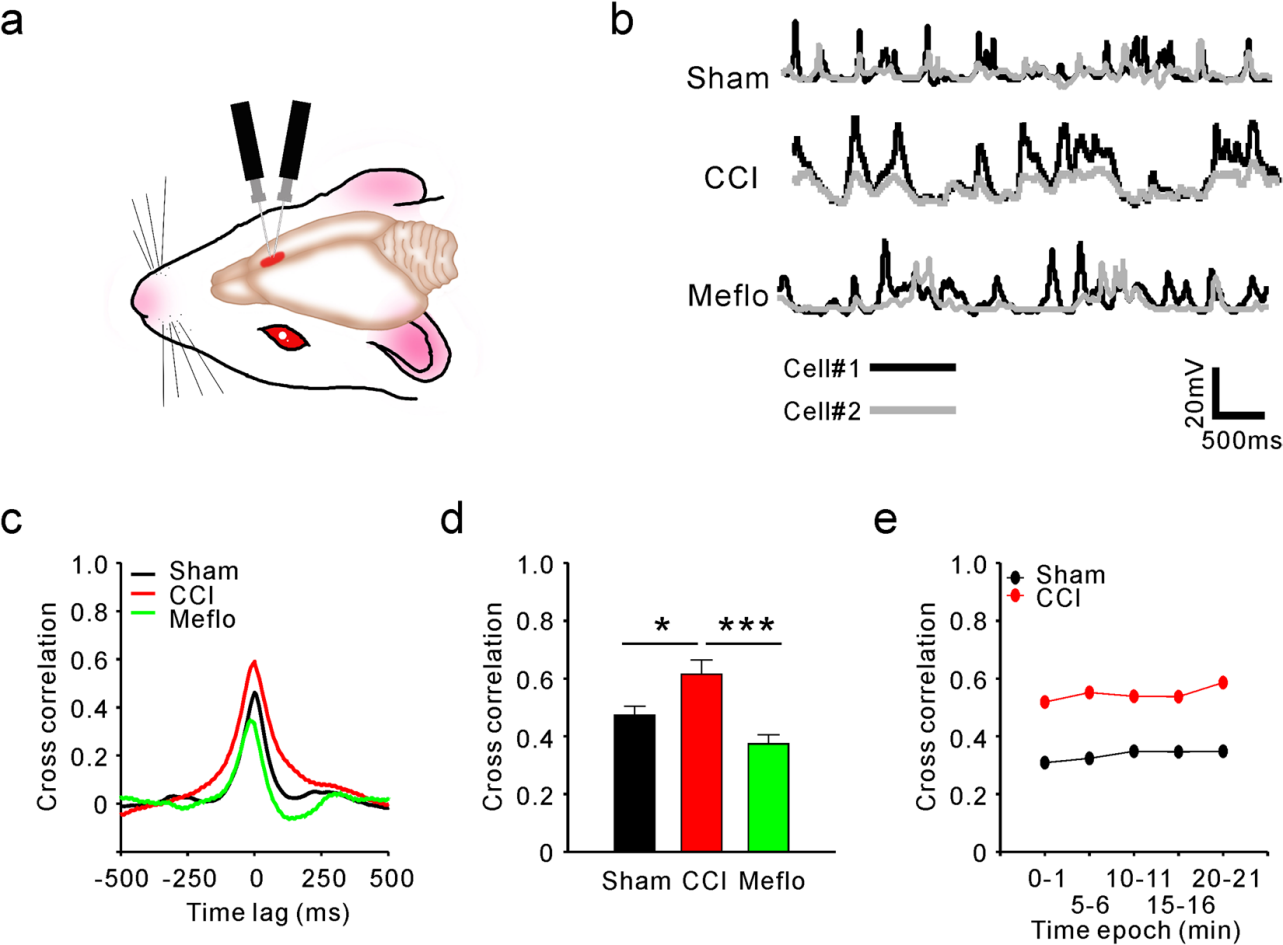
Cross-correlation of spontaneous membrane potential (V_m_) in the ACC neurons was increased continuously after CCI surgery, and this increase was reversed by Meflo. (**a**) Schematic diagram for *in vivo* dual patch clamp whole-cell recording from ACC neurons (**b**) Representative spontaneous V_m_ recorded in pairs of ACC neurons *in vivo* from different groups. Scale bars represent 20 mV and 500 ms. (**c**) Cross correlation of spontaneous V_m_ in pairs of ACC neurons from the sham, CCI and Meflo groups. A strong correlation was observed at zero time lag in the CCI group. Cross-correlation was increased in the CCI group compared with the sham group and decreased relative to the Meflo group. (**d**) Average correlation coefficients between spontaneous Vm in ACC neurons for the sham, CCI and Meflo groups (one-way ANOVA, *P < 0.05, CCI compared with the sham group; ***P < 0.001, compared with the Meflo group, n = 9). (**e**) Correlation coefficients in ACC neurons computed from the 1 min epoch differ little from coefficients computed from other 1 min recordings in the sham and CCI groups.

We recorded intracellularly from pairs of neurons in the ACC of anaesthetised rats (Fig. 2a). Electrophysiological recordings were performed within 1 to 2 weeks after CCI surgery. We recorded from 9 pairs of cells in each group, and the horizontal and vertical distance between cells in each pair was less than 500 µm. The whole-cell recordings from ACC neurons recorded *in vivo* indicated active spontaneous membrane potential fluctuations (Fig. 2b). Intracellular recordings showed that subthreshold fluctuations in membrane potential were correlated both in the CCI and sham groups (Fig. 2c). To quantify the extent of the synchrony between the cells in a pair from different groups, we computed the V_m_ cross-correlations and found that the V_m_ Correlation in CCI group was significantly stronger than in the sham group (0.47 ± 0.03 in Sham, and 0.62 ± 0.05 in CCI rats, P < 0.05) (Fig. 2d). To investigate whether the cells were synchronised continuously rather than for brief epochs, we calculated V_m_ correlation within a single pair of neurons for five different 1 min epochs. One single pair of neurons was randomly chosen from sham and CCI groups. Our data show that the V_m_ cross-correlation calculated from the 1 min epoch in ACC neurons differed little from that calculated from other 1 min records in the sham and CCI groups (Fig. 2e).

**Figure 3:**
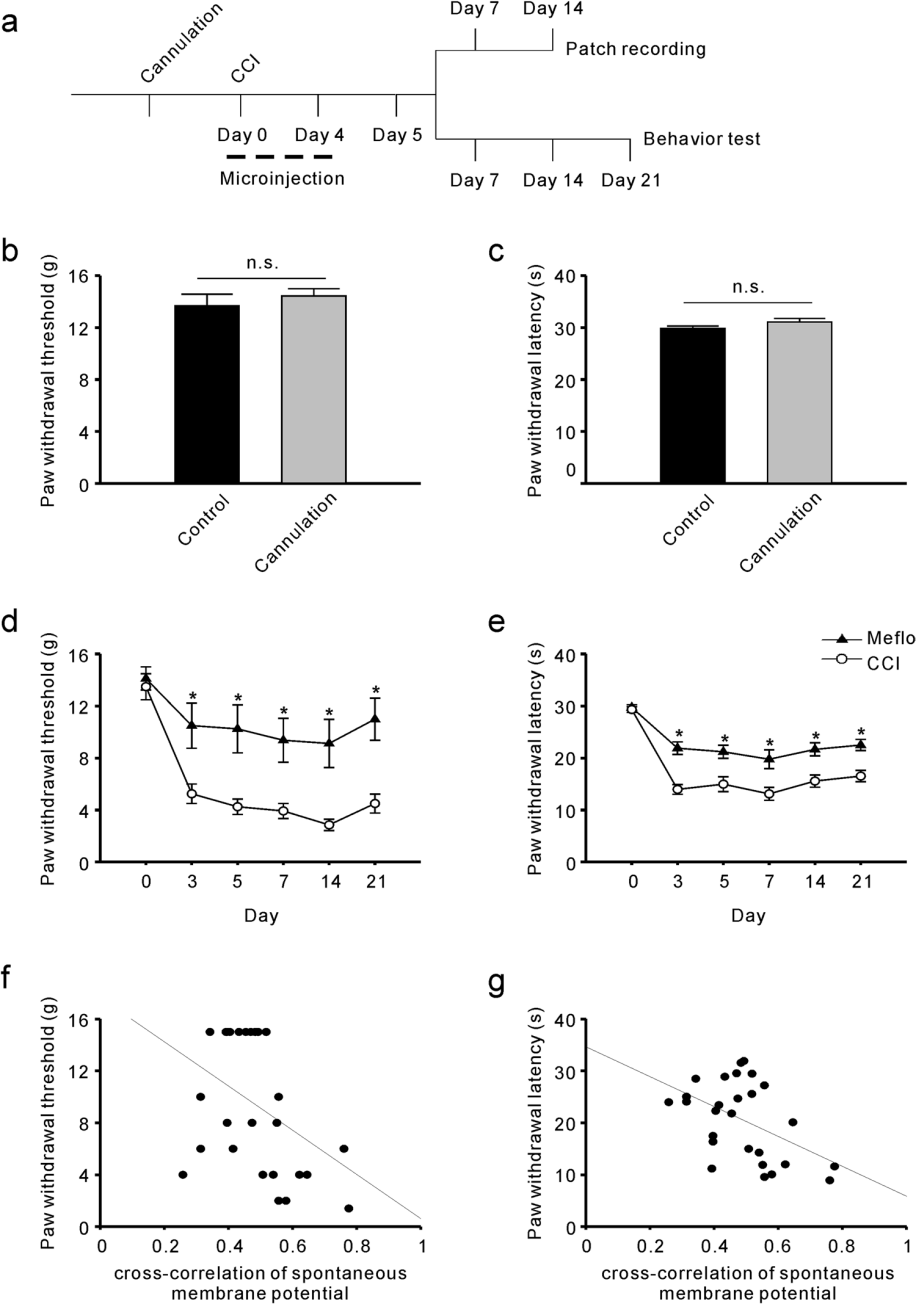
Meflo may relieve CCI-induced neuropathic pain. (**a**) Schematic view of cannulation and Meflo (100 mM, 6 μl) intracerebroventricular infusions. (**b**) Mechanical pain thresholds were not affected by cannulation (t-test, P = 0.54, n = 9). (**c**) Thermal pain thresholds were not affected by cannulation (t-test, P = 0.25, n = 9). (**d**) Reversal of mechanical allodynia after CCI by Meflo intracerebroventricular infusions (two-way ANOVA, *P < 0.05, compared with CCI group, n = 9). (**e**) Attenuation of CCI–induced thermal hyperalgesia by Meflo intracerebroventricular infusions (two-way ANOVA, *P < 0.05, compared with CCI group, n = 9). (**f**) V_m_ correlation was negatively correlated with the paw withdrawal mechanical thresholds of rats. (r = −0.408, P = 0.035). (**g**) V_m_ correlation was negatively correlated with the paw withdrawal thermal latencies of rats. (r = −0.478, P = 0.012).

To further confirm the associations between V_m_ correlation and neuropathic pain, we reversed the increase in V_m_ correlation in the CCI group and examined the resulting pain behaviour. Given that electrical synapses allow for many neurons to fire synchronously^18^ and have been reported to be responsible for generating neuronal oscillations^19,20^, we applied the electrical synapses blocker Meflo after CCI surgery and investigated the effects of Meflo administration on V_m_ correlation. We microinjected Meflo locally into the lateral ventricle after CCI, and the administration continued for the following 4 days. Dual patch clamp whole-cell recordings from ACC neurons in the Meflo group were recorded within 1 to 2 weeks after CCI. We additionally calculated V_m_ cross-correlations and found the V_m_ correlations were significantly decreased by Meflo (0.62 ± 0.05 in CCI rats, and 0.37 ± 0.03 in Meflo group, P < 0.001) (Fig. 2d). Furthermore, pain behaviours were also investigated in the Meflo group (Fig. 3a). We first observed whether cannulation will directly lead to changes in rat pain behavior and out data showed that cannulation did not significantly affect the mechanical (Fig. 3b) and thermal (Fig. 3c) pain thresholds of rats. Moreover, we found that Meflo (100 mM, 6 μl) was successful in attenuating mechanical allodynia (Fig. 3d) and thermal hyperalgesia (Fig. 3e) after Meflo intracerebroventricular infusions. To investigate the link between V_m_ Correlation and neuropathic pain behaviors, a comparison of V_m_ Correlation with the extent of pain behavior changes at individual animal level was done. A linear correlation analysis showed that V_m_ correlation was negatively correlated with the mechanical (r = −0.408, P = 0.035) (Fig. 3f) and thermal (r = −0.478, P = 0.012) (Fig. 3g) pain thresholds of rats.

### Power spectrum changes in membrane oscillations of different frequencies after CCI surgery

**Figure 4:**
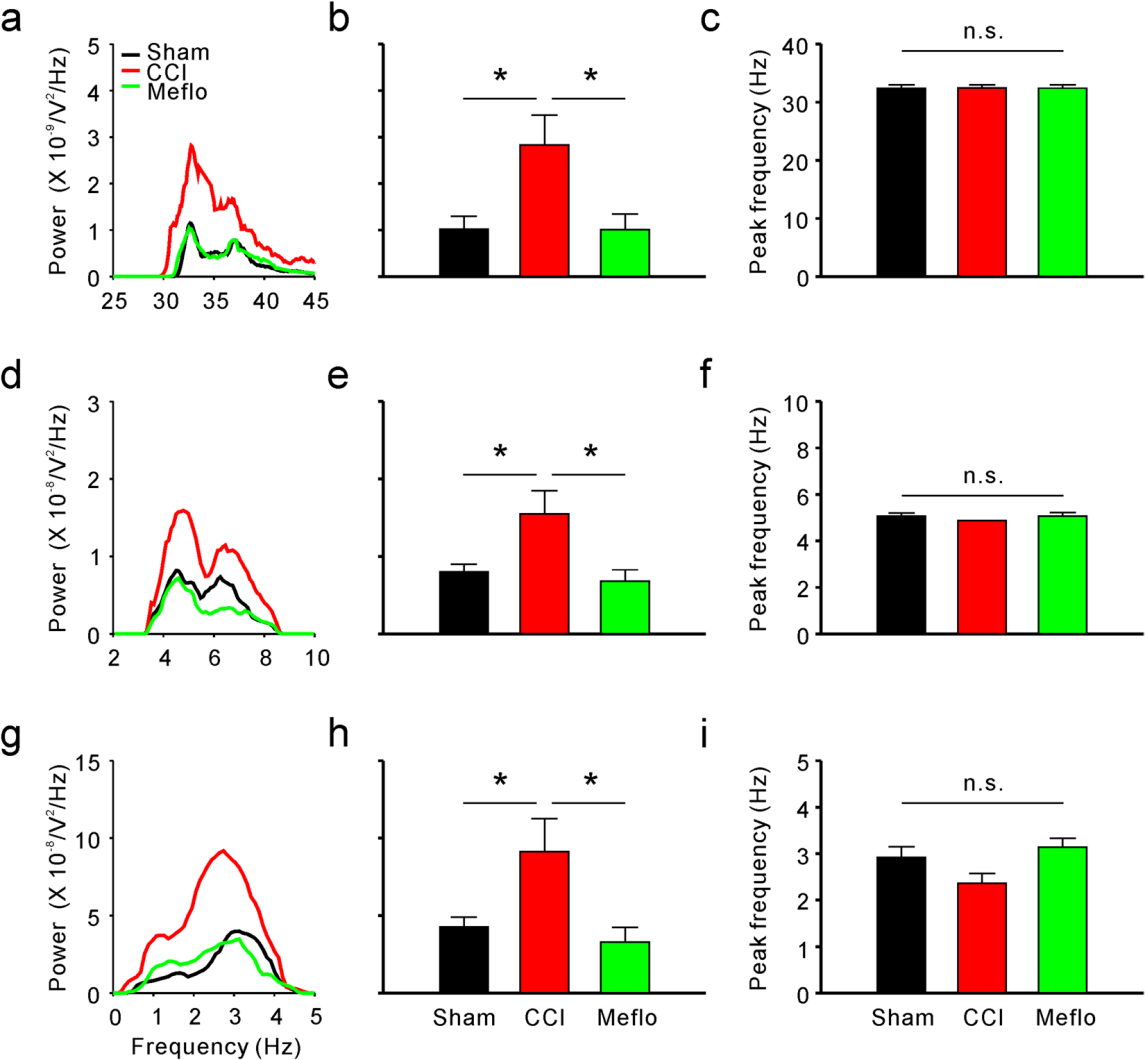
Nerve injury induces activation of higher-frequency (gamma rhythms) and lower-frequency (theta and delta rhythms) membrane potential synchrony in ACC neurons, and Meflo reverses activation induced by CCI surgery. (**a**) Power spectra of higher-frequency (gamma rhythms) membrane potential synchrony of neurons in the anterior cingulate cortex. (**b**) Summary results of the power of higher-frequency (gamma rhythms) membrane potential synchrony in ACC neurons from the sham, CCI and Meflo groups (one-way ANOVA, *P < 0.05, n = 18 for each group). (**c**) Peak frequency from the sham, CCI and Meflo groups (n = 18 for each group). (**d**) Power spectra of lower-frequency (delta rhythms) membrane potential synchrony of neurons in the anterior cingulate cortex. (**e**) Summary results of the power of lower-frequency (delta rhythms) membrane potential synchrony in the ACC neurons from the sham, CCI and Meflo groups (one-way ANOVA, *P < 0.05, n = 18 for each group). (**f**) Peak frequency from the sham, CCI and Meflo groups (n = 18 for each group). (**g**) Power spectra of lower-frequency (theta rhythms) membrane potential synchrony of neurons in the anterior cingulate cortex. (**h**) Summary results of the power of lower-frequency (theta rhythms) membrane potential synchrony in ACC neurons from the sham, CCI and Meflo groups (one-way ANOVA, *P < 0.05, n = 18 for each group). (**i**) Peak frequency from the sham, CCI and Meflo groups (n = 18 for each group).

**Figure 5:**
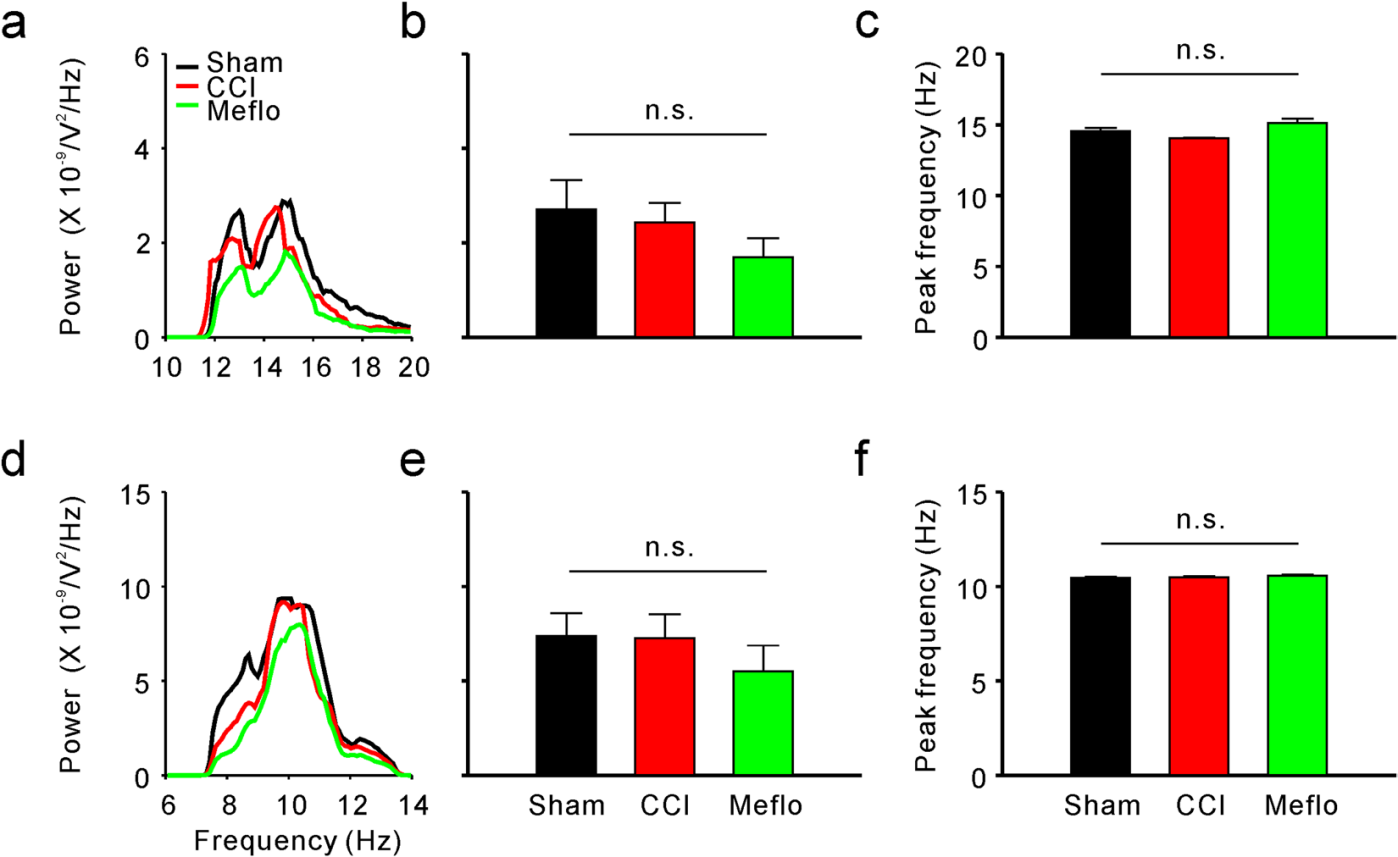
Nerve injury has no effect on middle-frequency (alpha and beta rhythms) membrane potential synchrony in the ACC neurons (**a**) Power spectra of middle-frequency (alpha rhythms) membrane potential synchrony of neurons in the anterior cingulate cortex. (**b**) Summary results of the power of middle-frequency (alpha rhythms) membrane potential synchrony in ACC neurons from the sham, CCI, and Meflo groups (one-way ANOVA, P = 0.52, n = 18 for each group). (**c**) Peak frequency from the sham, CCI, and Meflo groups (n = 18 for each group). (**d**) Power spectra of middle-frequency (beta rhythms) membrane potential synchrony of neurons in the anterior cingulate cortex. (**e**) Summary results of the power of middle-frequency (beta rhythms) membrane potential synchrony in ACC neurons from the sham, CCI, and Meflo groups (one-way ANOVA, P = 0.32, n = 18 for each group). (**f**) Peak frequency from the sham, CCI, and Meflo groups (n = 18 for each group).

It has often been proposed that membrane potential oscillations of different frequencies differ in function from each other. Thus, to examine the potential role of membrane potential oscillations of different frequencies in generating neuropathic pain, we additionally investigated the V_m_ fluctuations at different frequencies. Given that higher-frequency oscillations (gamma rhythms) have been previously associated with pain perception^10,11^, we first set out to analyse the gamma rhythms and found that the power of higher-frequency V_m_ fluctuations (gamma, 30–80 Hz) was significantly enhanced in the CCI group (1.02 ± 0.27 × 10^−9^ V^2^/Hz in the sham group and 2.83 ± 0.64 × 10^−9^ V^2^/Hz in CCI rats, P < 0.05) (Fig. 4a,b). The peak frequency of higher-frequency V_m_ fluctuations remained unaffected after CCI (Fig. 4c). Furthermore, lower-frequency (theta, 4–8 Hz and delta, < 4 Hz) oscillations are believed to be fundamental for the memory^21,22^. Given that neuropathic pain development and memory share similar mechanisms^23^, the lower-frequency oscillations may also play an important role in neuropathic pain pathogenesis. Thus, lower-frequency V_m_ fluctuations were also investigated (Fig. 4d,g). Similarly, there was no difference in the peak frequency of lower-frequency V_m_ fluctuations between the sham and CCI groups (Fig. 4f,i). Significant enhancements were also found in the power of lower-frequency V_m_ fluctuations after CCI surgery (theta: 0.80 ± 0.10 × 10^−8^ V^2^/Hz in sham rats and 1.55 ± 0.30 × 10^−8^ V^2^/Hz in CCI rats, P < 0.05; delta: 4.27 ± 0.62 × 10^−8^ V^2^/Hz in sham rats and 9.13 ± 2.13 × 10^−8^ V^2^/Hz in CCI rats, P < 0.05) (Fig. 4d,e,g,h). However, the power (Fig. 5a,b,d,e) and peak frequency (Fig. 5c,f) of middle-frequency V_m_ fluctuations (beta, 13–30 Hz and alpha, 8–13 Hz) remained unchanged after CCI surgery.

To further verify the relationship between the power spectrum of membrane oscillations and neuropathic pain, we also investigated the power of different frequencies of membrane potential synchrony at different frequencies in the Meflo group. The enhanced power of both higher-frequency (2.83 ± 0.64 × 10^−9^ V^2^/Hz in CCI rats and 1.01 ± 0.33 × 10^−9^ V^2^/Hz in Meflo group, P < 0.05) (Fig. 4a,b) and lower-frequency V_m_ fluctuations (theta, 1.55 ± 0.30 × 10^−8^ V^2^/Hz in CCI rats and 0.68 ± 0.14 × 10^−8^ V^2^/Hz in the Meflo group, P < 0.05; delta, 9.13 ± 2.13 × 10^−8^ V^2^/Hz in CCI rats and 3.30 ± 0.94 × 10^−8^ V^2^/Hz in the Meflo group, P < 0.05) (Fig. 4d,e,g,h) after nerve injury were reversed by intracerebroventricular Meflo infusions. Moreover, the Meflo had no effect on the power of middle-frequency V_m_ fluctuations or the peak frequency of V_m_ fluctuations (Fig. 5a,b,d,e).

### Similar Anaesthesia Level for Recordings in different groups

**Figure 6:**
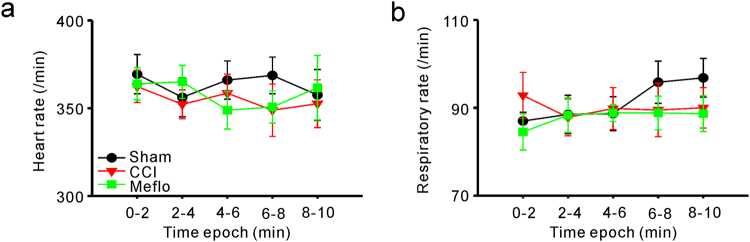
Similar anaesthesia levels for recordings in the sham, CCI and Meflo groups. (**a**) Summary of heart rates of all rats in different epochs from the sham, CCI and Meflo groups (two-way ANOVA, P = 0.95). (**b**) Summary of respiratory rates of all rats in different epochs from the sham, CCI and Meflo groups (two-way ANOVA, P = 0.84). No difference was detected in respiratory (**a**) and heart (**b**) rates between the sham, CCI and Meflo groups.

In the electrophysiological recordings, anaesthesia was maintained at the lightest possible levels for the sham, CCI and Meflo groups. To further preclude the possibility that observed difference in ACC activity between different groups resulted from the differences in anaesthesia levels, we monitored the respiratory and heart rates in all rats to evaluate anaesthesia levels. In the sham, CCI and Meflo groups, similar heart rates (two-way ANOVA, P = 0.95) (Fig. 6a) and respiratory rates (two-way ANOVA, P = 0.84) (Fig. 6b) were observed, indicating a similar level of anaesthesia for the 3 groups. Moreover, our data also showed that the heart rate (Fig. 6a) and respiratory rate (Fig. 6b) were stable during recording in different epochs for each animal. Thus, the higher cross-correlation between ACC neurons after CCI surgery was not due to differences in depth of anaesthesia between groups.

## Discussion

By recording membrane potentials (V_m_) from pairs of ACC neurons *in vivo*, we have shown that the correlation of V_m_ fluctuations between nearby cells significantly increased after CCI surgery. Moreover, we decreased the correlation of V_m_ fluctuations using Meflo and found that symptoms of neuropathic pain were subsequently relieved. Thus, our results may indicate that increases in V_m_ cross-correlations after nerve injury contribute to the generation of neuropathic pain. The occurrence of endogenous membrane synchrony was demonstrated in different portions of the central nervous system including the neocortex^24^, entorhinal cortex^25^, thalamus^26^, amygdaloid complex^27^, and inferior olive^28,29^. Here, we demonstrated the existence of the membrane synchrony in the ACC using *in vivo* dual patch whole-cell recording. According to previous research, many ACC neurons from layer II/III were activated after nerve injury^16^. Thus, we specially record ACC neurons from layer II/III. Observations of endogenous membrane synchrony in the central nervous system indicate that neural function is a cooperative process between neurons and synchronisation plays an important role in information processing in the brain. Although subthreshold oscillations do not directly result in neuronal firing, they assist in processing information from different sensory systems to form a consistent and unified perception of external stimuli^30,31^. Subthreshold oscillations were found to be an essential substrate of ectopic burst discharge^31^. Subthreshold membrane potential oscillations function as a modulator that determines at which moments synaptic input can have lesser or greater probability of triggering spiking. They may also facilitate synchronous activity of neighbouring neurons^32^. Previous studies have reported that subthreshold membrane potential oscillations play an important role in perception^6^, memory function^33^, and movement control^32^. However, to our knowledge, this is the first report of associations between neuropathic pain and subthreshold membrane potential synchrony of neurons in ACC. After nerve injury, the increased subthreshold membrane potential synchrony may facilitate synaptic transmission and greatly augment signals from external stimuli^31^. Thus, increased subthreshold membrane potential synchrony can be a major contributor to neuropathic pain and chronic pain.

What is the origin of the increased membrane potential synchrony that we observed? Spontaneous synchronised activity has been found to be organised locally through recurrent synaptic interactions^34^. Neurons in cortex are connected to more than one downstream neuron. Moreover, synaptic connections between the neurons are important for generating synchrony because strong synaptic connections may allow a burst in one neuron to evoke a membrane fluctuation in the follower neurons. Previous studies have demonstrated strong associations between electrical coupling and membrane potential synchrony. Knockdown of Cx36 blocked electrical coupling and reduced the synchrony^35^, amplitude, and continuity^20^ of subthreshold membrane potential oscillations. Our previous study has shown that electrical synaptic connections are significantly enhanced by CCI surgery. Electrical coupling mediates interactions between neurons and may give rise to increased membrane synchrony. In the current study, we applied the electrical synapses blocker Meflo and found that increased membrane synchrony was reversed by blocking electrical coupling. Thus, our data may further suggest that enhanced electrical synaptic connections after CCI may contribute to stronger membrane potential synchrony. Our previous data showed that the changes of electrical synapses in ACC area were mainly in the early stage after nerve injury (within 7 days) after nerve injury. Therefore, we use electrical synaptic blockers in the early post-nerve injury period. We found that blocking electrical synapses in the early phase after injury alleviated the degrees of pain behaviors throughout the time points for 3 weeks. Our results may indicate that early blockade of enhanced synchronized oscillations can lead to sustained pain relief.

Neural oscillation has been shown to be involved in pain perception and behaviour. A subset of neuropathic pain patients frequently exhibit abnormal synchronised oscillatory activity in the brain. By comparing power spectra in the EEGs of neuropathic pain patients and healthy controls, the patient group demonstrated significantly higher resting-EEG power in the theta frequency^36^. Moreover, the excessive theta power gradually decreased and returned to normal values after thalamic surgery^36^. In addition, patients with visceral^37^ and somatic pain syndromes^38,39^ also showed significantly higher power in delta and/or theta EEG oscillations compared with healthy controls. External stimuli could induce pain perception and activate gamma oscillations in pain-associated cortex^10^. The magnitudes of stimulus-induced gamma oscillations could always predict the subjective pain intensity^40^. In a previous study, we also found that gamma oscillations in the ACC play a key role in generation of neuropathic pain^12^. Previous studies on neural oscillations have used extracellular recordings; in contrast, in the current study, we obtained dual patch-clamp recordings of neighboring neurons in pain-associated cortex. Membrane potential fluctuations in the ACC can also oscillate at specific frequencies^6,32,41^. Frequency analyses revealed a broadband frequency distribution of V_m_ fluctuations in the ACC. Consistent with previous reports, we found enhanced power of both higher-frequency (gamma) and lower-frequency (theta and delta) V_m_ fluctuations after nerve injury compared with the sham group. Thus, higher- and lower-frequency membrane potential oscillations in the ACC may be related to processing of noxious inputs and play important roles in generating neuropathic pain. The lower-frequency membrane potential oscillations were mainly thought to be related to memory^21,22^, our results further confirm that pain and memory may share similar mechanisms. Moreover, middle-frequency oscillations were found to participate in modulation of motor behaviour^42^. Our results may indicate that neuropathic pain in the current animal model has little impact on modulation of motor behaviour in rats.

Changes in membrane potential synchrony in ACC neurons accompanying neuropathic pain were examined in the present study using whole-cell recording under anaesthesia, during which the awareness of pain may be largely abolished by the anaesthetics. Although we have found a similar depth of anaesthesia between groups, more aspects and/or different extents of neuropathic pain-associated changes in membrane potential oscillations may be observed in awake animal models.

## Materials and Methods

### Animals

Sprague–Dawley rats aged 9 to 11 weeks and weighing 240 to 340 g (Shanghai Sipper; BK Laboratory Animals Co., Ltd., China) were housed in cages at 24 °C and at 50 to 60% humidity with a 12/12 h light/dark cycle and a sufficient food and water supply. Rats were randomly divided into different groups. All experiments were performed under protocols approved by the Animal Care and Use Committee of East China Normal University and Shanghai Jiaotong University School of Medicine. All methods were performed in accordance with the relevant guidelines and regulations. All surgical procedures were performed under anaesthesia with intraperitoneal injection of pentobarbital sodium (40 mg/kg).

### Chronic constriction injury Surgery

The CCI model was established similarly to the method described by Bennett and Xie. Briefly, the left sciatic nerves were exposed and dissected from the surrounding connective tissue. Four chromic gut (5–0) ligatures were tied loosely around the nerve proximal to its trifurcation at 1-mm intervals. Sham surgery was performed by exposing the sciatic nerve without ligation. The muscle and skin were closed in layers using suture lines. All operations were performed by the same person.

### Cannulation and Microinjection

Animals were anaesthetised with pentobarbital sodium, and then secured on a stereotaxic apparatus. Burr holes were drilled over the lateral ventricle (1 mm posterior from bregma, +1.6 mm lateral to the midline) in accordance with the position determined by the atlas of Paxinos and Watson (1998). A 28-gauge guide cannula was implanted into the lateral ventricle (−3.6 ventral). Three stainless steel screws were implanted into the skull around the guide cannula and fixed with dental acrylic. Dummy cannulas were inserted after surgery and left in place until the infusion day. The animals were housed individually after surgery. Penicillin (80,000 units) was administered for infection prophylaxis 1 day before surgery and 2 days consecutively after surgery. Cerebral microinjection was performed 7 days after cannulation. The dummy cannulas were removed before microinjection. Internal injection cannulas (32 gauge), which extended 1 mm beyond the guide cannulas, were inserted. The solution was injected at a constant rate for a period exceeding 120 s using a microinjector. The injection cannulas were left in place for 3 additional min before being withdrawn. Once-daily, mefloquine (Meflo) was microinjected into the lateral ventricle on days 0, 1, 2, 3, and 4 after CCI.

### Behavioural studies

For mechanical allodynia tests, the animals were habituated for 2 to 3 days in the test environment prior to each test. Rats were placed in a plexiglass box with a metal net bottom for 30 min. After habituation to the environment, the hind paw was stimulated using one of a series of von Frey hairs with logarithmically increasing stiffness (0.6, 1, 1.4, 2, 4, 6, 8, 10, and 15 g) (Stoelting Co., USA), which was presented perpendicular to the plantar surface (5 to 6 s for each hair). A positive performance was recorded when the rat raised the hind limb escaping the pressure from von Frey hairs. The Dixon up–down method was used to determine the 50% withdrawal threshold. The experimenters who conducted the various assessments were blinded to the treatment conditions.

Thermal hyperalgesia was evaluated as the threshold of withdrawal responses to noxious heat stimuli, which was measured using a radiant heat method^43^. Briefly, rats were placed in a plexiglass box on a 3-mm-thick glass plate. After habituation in the box for 30 min, the sole skin of each animal was irradiated with light within a 0.5-cm-diameter circle using a BME-410 thermal radiation stimulator (Institute of Biomedical Engineering, Peking Union Medical College, Tianjin, China) at 10 V and 30 W. The time from irradiation initiation to paw withdrawal was recorded as the paw withdrawal latency value. A cutoff time of 40 s was used to avoid local burn injury. Three measurements were taken for each animal, with a 6- to 8-min interval allowed between trials, and the mean value was used for analysis.

### Electrophysiological recording

Before electrophysiological recording in CCI rats, hypersensitivity to mechanical stimulation, which developed together with hypersensitivity to thermal stimulation, was tested and found to be successfully induced in nearly all (>90%) of these rats. The CCI rats not showing this hypersensitivity were not considered for further recording. We specially record the anterior cingulate cortex (ACC) neurons from layer II/III^16^.

*In vivo* whole-cell recording was performed within 1 to 2 weeks after CCI surgery as described previously^44^. In brief, animals were anesthetized with pentobarbital sodium (initially with ~40 mg/kg; supplemented 2–4 h later with ~20 mg/kg/h; i.p.), and the procedure of anaesthesia was maintained at a light level just below the threshold of body movements consisting of licking or scratching. Rectal temperature was maintained at 37.3–37.8 °C using a heating blanket placed beneath the animal. To measure heart and respiratory rates, pressure sensors, customised with the use of piezoceramics, were placed under the chest and abdomen, respectively, and signals were sampled at 4 kHz with a data acquisition card (Digidata 1440, Axon Instr.). After tracheotomy, the head of the rat was restrained with a stereotaxic apparatus (David Kopf Instr.), and a hole (2–3 mm diameter) on the skull was drilled in the skull (0.3–0.6 mm lateral to the midline, 2–3 mm anterior to bregma) for recording. A small piece of dura mater was partially removed.

Patch pipettes with a tip opening of 1.5–2.0 μm were pulled from borosilicate glass tubing (Kimble Glass Inc.) with a resistance of 4.0–5.0 MΩ. Internal solution contained 140.0 mM of K-Gluconate, 2.0 mM K_2_ATP, 1.0 mM CaCl_2_, 2.0 mM MgCl_2_, 10.0 mM HEPES, 11.0 mM EGTA. The pH value of the internal solution was adjusted 7.3 with KOH. A positive pressure was applied to the pipette while it was advanced into the brain by a motor-driven manipulator (Siskiyou MMX7630, Siskiyou Corp.) at a speed of 15–30 μm/s. Signals were acquired with a patch-clamp amplifier (Axopatch 200B, Axon Instr.) and sampled at 5 kHz by a data acquisition card (Digidata 1440, Axon Instr.), with 1, 2, or 5 kHz low-pass filtering. In the neuron included for analysis, membrane resistance was 56 ± 5 MΩ, and series resistance (not compensated for) was 55 ± 11 MΩ. After the cell membrane was broken, we waited 10 min to ensure the stability of the cell state, followed by a continuous recording for 10 min. We divided this recording into 1-min intervals for data analysis, and the mean value was used for the final analysis. The 9 pairs of neurons recorded in 4–5 different animals of each group.

Cross-correlations in membrane potential changes were computed by subtracting the average value of each trace, normalising each trace to its standard deviation and then computing the correlation in IgorPro to generate a cross-correlogram with a maximal value of 1 for identical traces^45^. For power spectrum analysis of membrane potential fluctuations, all data were bandpass-filtered and divided into bins of <4 Hz, 4–8 Hz, 8–13 Hz, 13–30 Hz and 30–80 Hz. Peak frequency and power values in different frequency bands were obtained from power spectra generated using Fourier analysis in MATLAB software (MathWorks, USA). Max power values were summarized for statistical analysis.

### Statistical analysis

Neurons with resting potentials between −66 mV and −87 mV (−75 ± 0.5 mV; mean ± SEM) were considered for further analysis, and recordings with >15% changes in access resistance were further discarded. Liquid junction potentials (−13 mV) were corrected in this study. The data are expressed as the means ± SEM. A one-way ANOVA with Tukey’s post hoc tests for multiple comparisons were used when more than two groups of data were compared, and Student’s t test was used when only two groups were compared. Behavioural data were analysed with a two-way repeated-measures ANOVA including group as a between-groups factor and time points as a repeated-measures factor, followed by Bonferroni post hoc tests. To compare behavior results with V_m_ Correlation, a linear correlation analysis was done. The criterion for statistical significance was P < 0.05.

### Data availability statement

The datasets generated during and/or analysed during the current study are available from the corresponding author on reasonable request.
